# Resistant starch from five Himalayan rice cultivars and Horse chestnut: Extraction method optimization and characterization

**DOI:** 10.1038/s41598-020-60770-4

**Published:** 2020-03-05

**Authors:** Adil Gani, Bilal Ahmad Ashwar, Gazalla Akhter, Asir Gani, Asima Shah, Farooq Ahmad Masoodi, Idrees Ahmed Wani

**Affiliations:** 1 Department of Food Science and Technology, University of Kashmir, Srinagar, 190006 India; 2 Department of Food Technology, Faculty of Agro-Industry, Prince of Songkla University, Hat Yai, Songkhla 90112 Thailand

**Keywords:** Plant sciences, Chemistry

## Abstract

In this study resistant starch (RS) was extracted from five Himalayan rice cultivars and Indian Horse chestnut (HCN) using porcine pancreatin enzyme following which it was subsequently characterized for its physicochemical, structural and functional properties. *In vitro* digestibility test showed that RS content of the rice cultivars and HCN was in the range of 85.5 to 99.5%. The RS extracted from the rice cultivars and HCN showed significant difference in the apparent amylose content (AAC), ranging between 31.83 to 40.68% for rice and 45.79% for HCN. Water absorption capacity (WAC), swelling and solubility index of RS ranged from 112–133.9%, 5.28–7.25 g/g and 0.033–0.044 g/g, respectively. The rice RS granules were polyhedral and irregular shaped with granular length in the range of 4.8–5.9 µm. The HCN RS granule morphology showed smooth surfaced, round, elliptical, irregular and oval shapes with average granular length of 21 µm. Pasting behavior also varied significantly between rice RS and HCN RS with later showing the lower values of pasting properties. Thermal properties (T_0_, T_p,_ T_c_) and ΔH gel also varied considerably between the rice RS and HCN RS, wherein the highest values for peak gelatinization temperature and gelatinization enthalpy were seen for CH-1039. X-ray diffraction pattern of rice RS and HCN RS showed the characteristic A type of pattern in consonance with cereal starches.

## Introduction

The growing importance for functional foods has led to an intense awareness among individuals for healthy foods. Starch being one of the most important food components serves as an efficient and prime dietary source of energy contributing 60–70% of the total energy^1^. For nutritional purpose, starch is mainly classified into rapidly digestible starch (RDS), slowly digestible starch (SDS) and resistant starch (RS) depending upon its rate of digestion^2^. RS is one of the naturally occurring functional carbohydrates accounting to about more than 30% of the fiber content in foods^3^. RS is defined as the sum of starch and its degradation products which escapes the normal route of digestion but undergoes fermentation by the intestinal microflora, thereby results in the formation of short chain fatty acids (SCFA) such as acetic acid, butyric acid and propionic acid that are thought to protect against large bowel cancer besides possessing varied health benefits to the colon by reducing pH and increasing regularity^4^. The functional properties of RS has been acknowledged for the control of lifestyle syndromes like obesity, diabetes and subsequently, for reducing the risk of cardiovascular diseases^5^. RS is further sub classified into four major types based on its source and processing conditions^2^: RS1, RS2, RS3 and RS4. RS1 is physically inaccessible or entrapped starch and is mainly confined to the tough coatings of whole unprocessed cereal grains and legumes. RS2 is inaccessible to enzymatic action due to its crystalline nature such as high amylose maize starch (HAM). Both RS1 and RS2 belong to the category of native RS. RS3 is formed when starch rich foods are cooked and cooled during food processing applications by which the starch undergoes gelatinization and subsequent retrogradation thereby rendering the starch less soluble. RS4 refers to the category of starches that are chemically modified by various techniques to resist the normal score of digestion process such as starch esters, cross linked starches etc^2^. Rice (*oryzea sativa*) serves as an important and prime dietary source to over half of the world’s population. Rice with its high starch content is of particular interest for extraction of RS as it will implement in the dietary prevention of obesity, diabetes and cardiovascular diseases, similarly Indian horse chestnut (*Aesculus indica*) with about 38.3% starch content^6^ also serves as an important source for extraction of RS.

Though several researchers have developed the RS from rice and HCN starches by using physical, chemical and enzymatic modifications, no systematic study has been done so far on the extraction of natural RS from rice and HCN. Therefore, the objective of this study was to extract and characterize the natural RS from the five Himalayan rice cultivars and HCN.

### Experimental setup

#### Materials

Five rice cultivars viz. SK-46, SK-338, SK-406, CH-1039 and CH-1007 were procured from SKUAST-K. The seeds of Indian Horse Chestnut (HCN) were harvested from the trees of Botanical garden, Department of Botany, University of Kashmir, Hazratbal, Srinagar, India, 190006. Seeds were dehulled and stored at refrigeration temperature until further use. Glucose oxidase/peroxidase reagent (GOPOD) was purchased from Megazyme International, Wicklow, Ireland. Pancreatin, amyloglucosidase and α-amylase were obtained from Sigma-Aldrich, St. Louis, USA. All other reagents used in the study were of analytical grade.

#### Starch extraction

Starch was extracted from five rice cultivars and HCN by the alkaline steeping method^7^.

#### Resistant starch extraction

Briefly 4 g starch (dry weight basis) and 160 mL of enzyme mixture were added, mixture was vortexed and incubated in a shaking water bath 37 °C for 16 h (200 strokes/min) to hydrolyze the digestible starch (Enzyme mixture was prepared by suspending 3.2 g pancreatin in 320 mL of 0.1 M sodium acetate buffer, pH 5.2. Then 3.2 mL of amyloglucosidase was added). At the end of incubation period, the suspension was mixed with absolute ethanol (160 mL) and vortexed to deactivate the enzymes. RS was recovered as a pellet by centrifugation (1,500 × g, 10 min). Pellet was washed twice with 50% ethanol to remove the digested starch. The RS was dried in an oven at 40 °C.

#### Proximate composition

The extracted RS was analyzed for moisture (925.10), protein (920.87), fat (920.85) and ash (923.03) according to the methods of AOAC, 1990^8^.

#### Apparent amylose content

Apparent amylose content (AAC) of RS samples was determined by the colorimetric method^9^.

#### Resistant starch content

RS content was determined by the approved AACC method 32–40 (AACC, 2000)^10^. Briefly 4 mL of α-amylase (10 mg/mL) containing amyloglucosidase (3 U/mL) was added to 100 mg of each RS sample, mixture was vortexed followed by incubation at 37 °C in a shaking water bath (200 strokes/min) for 16 h to hydrolyze digestible starch. Then absolute ethanol (4 mL) was added to deactivate the enzymes, followed by centrifugation at 1,500 × g for 10 min. The pellet obtained was washed twice with 50% ethanol to remove the digested starch. The sediment was dissolved in KOH (2 mL, 2 M) by vigorous stirring for 20 min. The solution was neutralized with sodium acetate buffer (8 mL, 1.2 M). Then 0.1 mL of amyloglucosidase (3300 U/mL) was added followed by incubation for 30 min at 50 °C. The samples were centrifuged at 3000×g for 10 min. GOPOD (3 mL) was added to aliquots (0.1 mL) of the supernatant and incubated at 50 °C for 20 min. Absorbance was measured at 510 nm using a spectrophotometer. RS was calculated as the amount of glucose × 0.9.

#### Swelling & solubility index

Swelling & solubility index was determined according to Ashwar *et al*.^8^.

#### Water absorption capacity

Water absorption capacity (WAC) of RS was determined according to Ashwar *et al*.^8^.

#### Pasting properties

Pasting properties of RS samples were measured using a Rapid Visco Analyzer (Tech Master, Pertain Instruments Warriewood, Australia). RS was dispersed in water (14% moisture basis, 28 g total weight). The dispersion was equilibrated at 50 °C for 1 min, followed by heating to 95 °C at the rate of 12.2 °C/min, held at 95 °C for 2.5 min, cooled to 50 °C at the rate of 11.8 °C/min and again held at 50 °C for 2 min. A rotating paddle (160 rpm) was used throughout the entire analysis, except for rapid stirring (960 rpm) for the first 10 s to disperse the sample.

#### Thermal properties

The thermal properties of RS samples were studied with a Differential Scanning Calorimeter (DSC-1 STAR-System, Mettler-Toledo). 3.5 mg of RS sample and 8 µL of deionized water were weighed in a platinum pan. The sample was heated from 20 to 200 °C at 10 °C/min. An empty platinum pan was used as a reference.

#### Scanning electron microscopy

The RS samples were placed on an adhesive tape attached to an aluminum specimen stub. After coating with gold-palladium, the samples were scanned at 5 kV in a scanning electron microscope (Hitachi S-300H-Tokyo, Japan).

#### Attenuated Total Reflectance- Fourier Transform Infrared (ATR-FTIR) Spectroscopy

Spectra of RS samples were recorded using ATR-FTIR system (Cary 630 FTIR, Agilent Technologies, USA) in the range of 4000–400 cm^−1^ at room temperature.

#### X-ray diffraction

X-ray diffraction pattern of the RS samples was recorded using X-ray diffractometer (X’Pert PRO, Panalytical, Netherlands) with a wavelength of 0.154 nm. The diffractometer was operated at 40 kV and 35 mA. The diffractograms were obtained at 25 °C over a 2 Ø range of 4–40° with a step size of 0.02 and sampling interval of 10 s. The relative crystallinity (RC) of RS samples was calculated by the equation:

$${\rm{RC}}( \% )=({\rm{Ac}}/{\rm{Ac}}+{\rm{Aa}})\times 100$$

where Ac is the crystalline area and Aa is the amorphous area on the X ray diffractograms.

### Statistical analysis

Data reported are the averages of triplicate observations. Analysis of variance (ANOVA) with a significance level of 5% was applied and Duncan’s test was used to determine the differences between means with the help of commercial statistical package (SPSS 16.0).

## Results and Discussions

### Proximate composition

Moisture content of the RS samples ranged from 4.20% (SK-406) to 6.73% (CH-1007) (Table 1). The low moisture content of RS samples was probably due to the removal of pentosans and water binding proteins during alkali extraction. Protein, fat and ash contents of RS samples varied from 0.04–0.44%, 0.00–0.45% and 0.21–0.87% respectively. The lower values of protein content indicated that the RS were pure and the extraction method was effective in isolation and purification of RS.

**Table 1:** Proximate composition of rice and horse chestnut resistant starches (n = 3).

Resistant starch	Moisture (%)	Protein (%)	Fat (%)	Ash (%)
HCN	4.22^a^ ± 0.65	0.04^a^ ± 0.04	0.00^a^ ± 0.00	0.21^a^ ± 0.03
SK-46	5.53^b^ ± 0.65	0.44^b^ ± 0.02	0.42^d^ ± 0.02	0.78 ^cd^ ± 0.08
SK-406	4.20^a^ ± 0.36	0.44^b^ ± 0.03	0.45^d^ ± 0.03	0.85^de^ ± 0.03
SK-338	4.53^a^ ± 0.40	0.27^a^ ± 0.04	0.31^b^ ± 0.02	0.72^c^ ± 0.01
CH-1039	6.63^c^ ± 0.56	0.44^b^ ± 0.03	0.41^b^ ± 0.02	0.58^e^ ± 0.29
CH-1007	6.73^c^ ± 0.15	0.41^b^ ± 0.03	0.36^b^ ± 0.04	0.87^b^ ± 0.03

Values expressed are mean ± standard deviation.

Means in the columns with different superscripts are significantly different at p ≤ 0.05.

### Apparent amylose content (AAC) and resistant starch content (RS)

The AAC of the rice RS samples varied significantly (p ≤ 0.05) with the values ranging from 31.83% (SK-338) to 40.68% (CH-1007), however the RS extracted from HCN showed the highest value of AAC (45.79%) (Table 2). The AAC of the native rice starch ranged from 13.75% to 20.91%^11^ and the AAC of native HCN was found to be 30.15^12^. The huge increase in AAC of RS samples might be due to the action of amyloglucosidase and amylase enzymes on the α-(1–6) linkages of amylopectin molecules during the extraction of RS^13^. Similar inferences were reported in literature^14^. However higher amylose content (46.78–50.18%) was reported in resistant starch of elephant foot yam^15^. Differences in the amylose content of different rice starches have been reported to be influenced by the botanical source of starch, climatic and agronomic conditions, cultivar, starch isolation procedures and analytical methods^16,17^. The activity of the enzymes involved in starch biosynthesis may also be responsible for the variation in amylose content among the various starches^18^.

**Table 2:** Physicochemical properties of rice and horse chestnut resistant starches (n = 3).

Resistant starch	Amylose (%)	WAC (%)	Resistant Starch (%)	Swelling (g/g)	Solubility (g/g)
SK-46	37.9^b^ ± 1.43	112^b^ ± 2.82	92.75^b^ ± 0.99	5.28^b^ ± 0.32	0.033^b^ ± 0.06
SK- 406	36.95^b^ ± 2.55	113.9^a^ ± 4.17	89.72^b^ ± 0.87	5.59^b^ ± 0.81	0.035^ab^ ± 0.00
SK- 338	31.83^a^ ± 1.38	114.3^a^ ± 2.07	85.54^a^ ± 0.83	7.25^d^ ± 0.12	0.037^ab^ ± 0.01
CH -1039	32.12^a^ ± 3.62	112.4^b^ ± 2.47	90.03^b^ ± 1.03	6.55^c^ ± 0.32	0.044^b^ ± 0.06
CH-1007	40.68^b^ ± 1.98	113.5^a^ ± 2.54	90.97^b^ ± 1.68	5.56^b^ ± 0.02	0.033^ab^ ± 0.00
HCN	45.79^c^ ± 1.87	133.9^c^ ± 5.30	99.95^c^ ± 0.99	4.72^a^ ± 0.06	0.039^a^ ± 0.00

Values expressed are mean ± standard deviation.

Means in the columns with different superscripts are significantly different at p ≤ 0.05.

The RS contents (%) of five rice cultivars also varied significantly (p ≤ 0.05) with the values ranging from 85.54% (SK-338) to 92.75% (SK-46), however HCN showed the highest value for RS content (99.5%). Higher RS contents of CH-1007, SK-46 and HCN were due to their higher amylose contents. Several reports also showed a positive correlation between the amylose and RS contents^8,19^. Besides AAC, various other factors were suggested to affect RS content like molecular association between starch components, degree of crystallinity, and amylose-lipid complexes^20^.

### Swelling and solubility index

The swelling power indicates the capacity of the starch granules to uptake water at elevated temperature. Starch granules with greater crystalline areas along with strong covalent interactions in the crystalline regions usually show little swelling power in cold water than subsequent heating^20^. The gels formed from these crystalline regions are weak and have greater tendency towards retrogradation because of the covalent bonds^15^. The swelling power of rice RS ranged from 5.28 g/g (SK-46) to 7.25 g/g (SK-338) whereas solubility index ranged from 0.033 g/g (SK-46 and CH-1007) to 0.040 g/g (CH-1039) (Table 2). The swelling power of RS samples with higher amylose contents was significantly (p ≤ 0.05) lower than RS samples with lower amylose contents. Our results were in close agreement with the observations reported in literature^21–23^, wherein the swelling power of glutinous rice with higher amylopectin content was more pronounced than long grain rice with higher amylose content. HCN RS showed the lowest value for swelling index (4.72 g/g) which could be probably due to its highest amylose content, as swelling is primarily a property of amylopectin^21,24^ plus some water absorbing pentosans that may have remained during the extraction process. The swelling and solubility index was found to be influenced by various factors like variation between different cultivars, topographical conditions, starch isolation methods, degree of debranching, length of chains, conformation of the molecules and the ratio of amylose and amylopectin molecules^25–27^.

### Water absorption capacity

Water absorption capacity represents the ability of the starch molecules to associate with limited water addition. Among the five rice RS samples, SK-338 showed the highest value of WAC (114.3%) whereas SK-46 showed the lowest value (112%) (Table 2). The highest value of WAC in SK-338 could be due to lower soluble amylose content^21^ and intensive association of hydroxyl groups in between starch chains resulting in the formation of covalent and hydrogen bonds^28^. However HCN showed the highest value of WAC (133.9%) among all the RS samples. The highest WAC value of HCN may be due to its highest amylose content that results in higher water uptake and loose compaction of amylose and amylopectin^29^. The WAC of starch is greatly dependent on the source, content of amylose/amylopectin, extraction procedure and thermal stability^30,31^.

### Pasting properties

Pasting properties indicates the behavior of starch granules that ultimately determines the functionality and cooking quality of starch^32^. The pasting property plays a pivotal role in the application of starch and resistant starch in food industries and these parameters are dependent on the source, amount of starch, interaction between starch molecules and testing conditions^33^. Pasting properties of RS samples are given in Table 3. Peak viscosity varied significantly (p ≤ 0.05) between the RS samples, with CH-1007 showing the highest value of 1152 cP and SK-338 showing the lowest value of 842.5 cP among the rice RS. Among all the samples HCN showed the lowest peak viscosity value of 539.5 cP. Low peak viscosity of RS samples may be due to higher values of amylose contents that tends to decrease the melting temperature of the starch granules by disrupting their crystallinity. Our results are in agreement to the previous findings that showed low amylose starches have higher peak viscosities and also attain their peak viscosities at a lower temperature than high amylose starch samples^34–37^. In case of HCN, the reduction in the swelling index of RS as well as higher amylose content could attribute to lesser peak viscosity. Amylose is shown to inhibit swelling of starch granules that resulted in lower peak viscosity at higher temperatures^14,38^. Pasting temperature is the temperature at which the viscosity of the starch pastes begins to rise. Pasting temperature of rice RS was found in the narrow range of 94.95 °C–95.14 °C. However HCN RS showed significantly (P ≤ 0.05) lowest value of pasting temperature (70.07 °C). Trough viscosity of rice RS ranged from 636 cP (SK-338) to 888.5 cP (SK-406), however trough viscosity was considerably lower for HCN (180.5 cP). Final viscosity of rice RS ranged from 1412 cP (SK-46) to 2021.7 cP (CH-1007), however HCN RS showed lowest final viscosity (239) cP. The final viscosity is the measure of the ability of the starch to form a viscous paste and is because of the re-association of amylose and amylopectin molecules^39^. Lowest breakdown viscosity was seen for SK-46 (169.0 cP) and highest for CH-1007 (482.5 cP), similarly setback viscosity was seen to range from 636 cP (SK-338) to 1351 cP (CH-1007). Due to low amylose content in SK-46 and SK-338, breakdown and setback viscosities were substantially low; similarly the highest setback in CH-1007 was attributed to its highest amylose content. The disintegration of swollen starch granules results in BD (breakdown), while the variations of BD among rice starches may be attributed to the differences in rigidity of swollen granules. SB shows the extent of recovery of starch viscosity during cooling of the heated starch pastes, and is usually related to the AAC of starch^40,41^. The pasting properties of the rice and Horse chestnut resistant starches were lower than their corresponding native starches^11,42^ that could be because of the enzymatic hydrolysis of starch which includes increased formation of short linear chain molecules and increased RS content which could be substantiated to the decrease in the pasting viscosity along with the reduced ability of forming gels^22,43^.

**Table 3:** Pasting, thermal properties and % crystallinity of rice and Horse chestnut resistant starches.

Parameter	SK46	SK 338	CH 1007	CH 1039	SK 406	HCN
**Pasting properties**						
Peak viscosity (cP)	847^b^ ± 4.24	842.5^b^ ± 9.19	1152.5^e^ ± 3.53	1030^c^ ± 11.31	1088^d^ ± 16.97	539.5^a^ ± 12.02
Trough viscosity (cP)	677.5^c^ ± 10.60	636.5^b^ ± 4.94	670^c^ ± 19.79	736.5^d^ ± 4.94	888.5^e^ ± 10.60	180.5^a^ ± 9.19
Breakdown viscosity (cP)	169.0^a^ ± 14.84	206^b^ ± 4.24	482.5^e^ ± 7.07	293.5^c^ ± 6.36	199.5^b^ ± 12.13	359^d^ ± 2.82
Final viscosity (cP)	1412^c^ ± 11.31	1272.5^b^ ± 10.26	2021^e^ ± 16.26	1422.5^c^ ± 9.19	1540^d^ ± 9.89	239.5^a^ ± 6.36
Setback viscosity (cP)	734.5^d^ ± 21.92	636^b^ ± 14.5	1351^e^ ± 12.72	686^c^ ± 14.14	651.5^b^ ± 0.70	59.0^a^ ± 18.38
Peak time (min)	7.0^b^ ± 0.00	7.0^b^ ± 0.00	7.0^b^ ± 0.00	7.0^b^ ± 0.00	7.0^b^ ± 0.00	3.14^a^ ± 0.01
Pasting temperature (°C)	94.95^b^ ± 0.19	95.145^d^ ± 0.07	95.00^c^ ± 0.16	95.14^d^ ± 0.07	94.95^b^ ± 0.19	70.72^a^ ± 0.03
**Thermal properties**						
T_0_ (°C)	39.15^a^ ± 2.97	38.75^a^ ± 3.69	40.98^b^ ± 3.42	46.87^b^ ± 3.54	38.91^a^ ± 4.023	40.89^b^ ± 2.29
T_P_ (°C)	74.21^ab^ ± 1.78	73.87^ab^ ± 1.78	73.46^a^ ± 4.80	78.40^ab^ ± 3.47	76.66^b^ ± 3.18	75.23^b^ ± 1.78
T_C_ (°C)	107.87^b^ ± 2.10	106.72^b^ ± 5.86	116.12^b^ ± 3.72	94.05^a^ ± 2.60	110.00^b^ ± 4.05	121.06^b^ ± 2.60
T_C_-T_0_ (°C)	68.72b^c^ ± 1.86	67.97^bc^ ± 0.84	75.14^bc^ ± 0.24	50.18^b^ ± 19.55	72.94^bc^ ± 4.41	80.17^c^ ± 9.80
∆H gel (J/g)	131.45^e^ ± 3.85	130.0^e^ ± 4.22	146^d^ ± 4.63	370.87^a^ ± 5.48	186.04^b^ ± 7.19	161.63^c^ ± 9.21
% Crystallinity	17.72^b^ ± 0.92	17.12^b^ ± 1.00	17.38^b^ ± 0.89	17.11^b^ ± 0.97	16.75^b^ ± 1.05	13.90^a^ ± 1.05

Values expressed are mean ± standard deviation.

Means in the columns with different superscripts are significantly different at p ≤ 0.05.

### Thermal properties

Thermal properties of rice and Horse chestnut RS samples are presented in Table 3. The gelatinization temperatures (T_0_, T_p_ and T_c_) and enthalpy of gelatinization (ΔH_gel_) varied significantly (p ≤ 0.05) between the rice and Horse chestnut RS. In general CH-1039 and SK-406 showed the highest peak gelatinization temperature while as the lowest value was shown by SK-338, probably because of its lowest amylose content. Previous reports have also related the gelatinization temperature of starch to its amylose content^44,45^. The highest peak gelatinization temperature of CH-1039 recorded by the DSC was also consistent with its high pasting temperature obtained from the RVA data. CH-1039 shows highest value for ΔH_gel_ (370.87 J/g). This increase in the enthalpy of gelatinization has been attributed to the compact physical structure because of lower amylose and the higher quantity of amylopectin as it forms a dense, highly ordered structure^46^ hence more energy is needed to disrupt intermolecular hydrogen bonds linking adjacent chains or helices within starch structure^47^. The differences in T_0_, T_p_ and T_c_ among the rice resistant starches may be attributed to differences in AAC, granular architecture, molecular weight distribution and structure of amylopectin molecules^48^. The T_0_, T_p_ and T_c_ of HCN were 40.89, 75.23 and 121.06 °C. The high gelatinization temperature of HCN might be because of its higher AAC, wherein amylose restricted the hydration of amorphous regions, hindered the gelatinization of crystalline areas and raised the gelatinization temperature.

### Morphology

**Figure 1:**
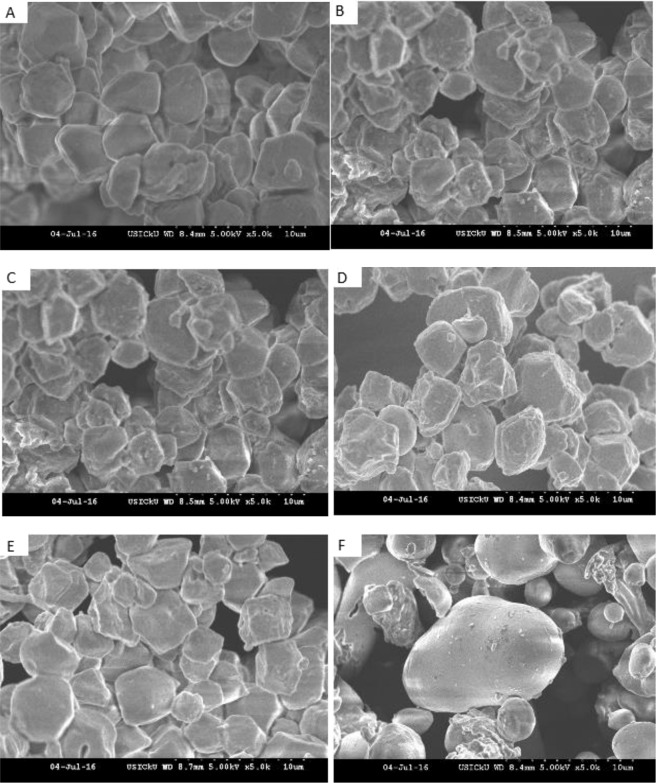
SEM images of resistant starches (**A**) SK-46, (**B**) SK-338, (**C**) SK-406, (**D**) CH-1039, (**E**) CH-1007 and (**F**) HCN.

Scanning Electron Microscopy (SEM) images of the rice and HCN resistant starches are shown in Fig. 1. The rice RS granules were polyhedral and irregular shaped with granular length in the range of 4.8–5.9 µm. The HCN RS granule morphology showed smooth surfaced round, elliptical, irregular and oval shapes with average granular length of 21 µm. Similar results are reported for starch isolated from Himalayan rice cultivars^42^. The morphology of starch granules depends on the biochemistry of chloroplast or amyloplast, as well as the physiology of plant^48^. Some reports claimed that the variation in size and shape of starch granules of different plant sources is attributed to biological origin^49^, however several other studies suggested that starch biosynthesis results in natural variability in amylose and amylopectin molecules that might be responsible for diversity of starch granules^50^. Other factors responsible for starch granule diversity are climatic conditions, agronomic factors and processing^51^.

### ATR-FTIR

**Figure 2:**
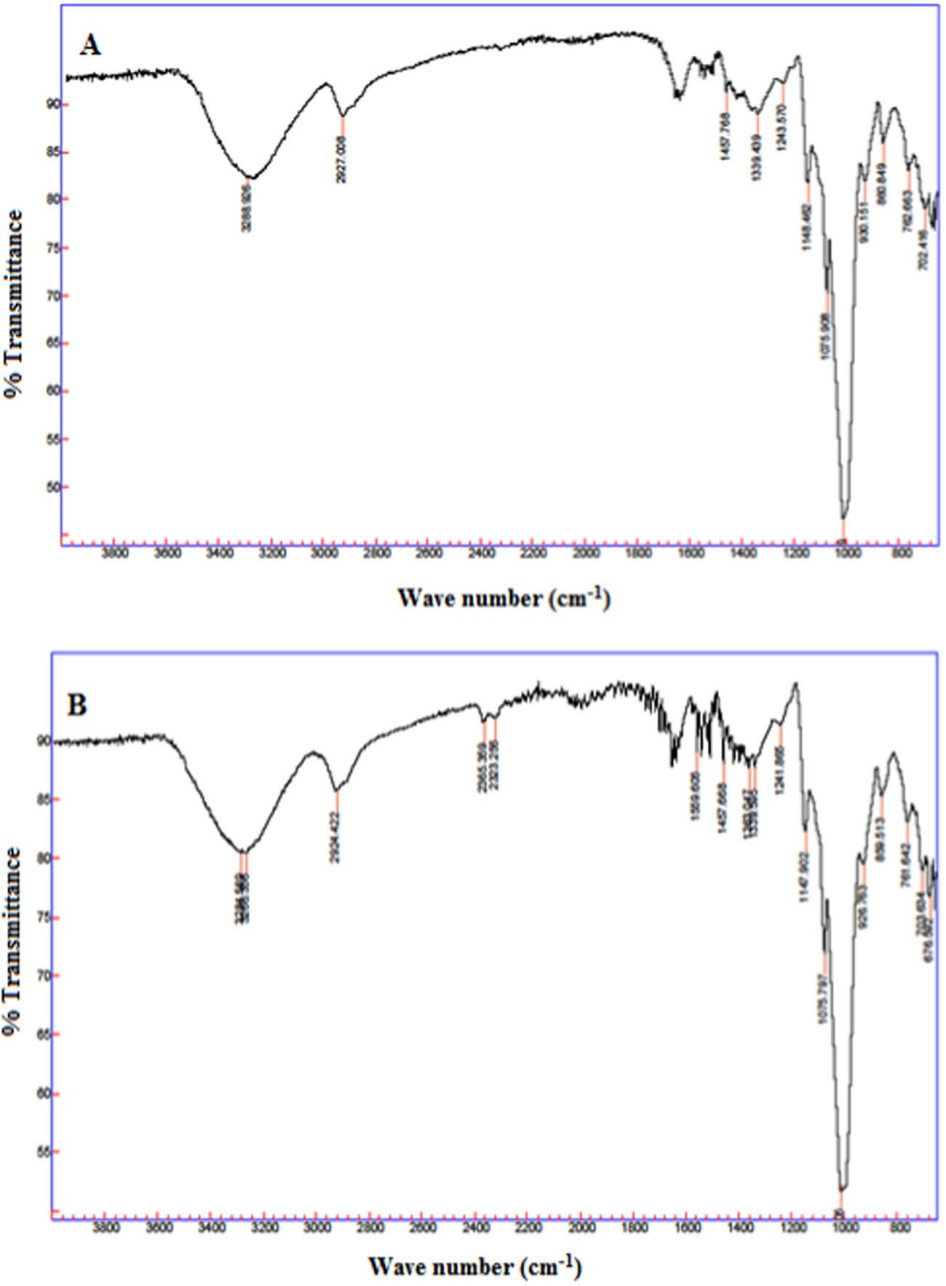
FT-IR of resistant starches: (**A**) rice starch, (**B**) HCN.

The FTIR spectrum of the rice and horse chestnut RS samples is shown in Fig. 2. HCN and rice RS showed absorption bands ranging from an extremely broad band at 3284–3288 cm^−1^ attributed to hydrogen bonded O-H stretching vibration; the sharp band ranging from 2924–2927 cm^−1^ attributed to C-H bond stretching associated with the ring methane hydrogen atoms; the band ranging from 1559–1650 cm^−1^ ascribed to H-O-H bending vibration; the band from 1339–1363 cm^−1^ attributed to the bent modes of O-C-H, C-C-H, and C-O-H, the bands at 1075 cm^−1^ and 1147–1148 cm^−1^ had both been assigned as the coupling of C-O, C-C and O-H bonds and the band at 1175–1140 cm^−1^ has been assigned to the C-O-C glycosidic bond^52^. The C-O-H bending modes observed at 1047 and 1022 cm^−1^ have been used to express the crystalline and amorphous structures of starch, respectively^8,20,53,54^. The FTIR analysis of HCN RS showed close results with the reported values for native horse chestnut starch^12^. There was also no remarkable band difference among the RS extracted from five rice cultivars suggesting that the basic structure of RS samples is similar which is being reflected in their similar absorption bands.

### X-ray diffraction

**Figure 3:**
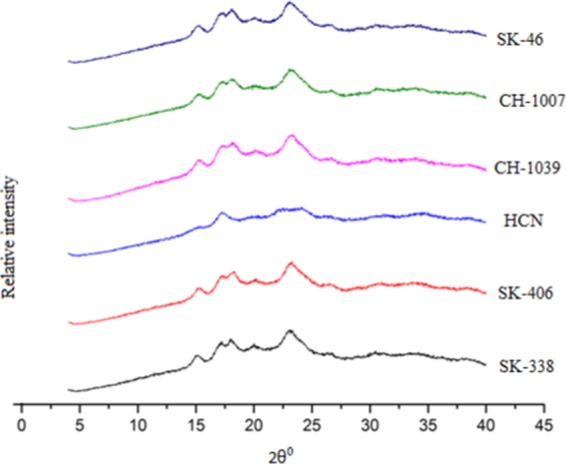
XRD pattern of Resistant starches.

The X-ray diffraction patterns of rice and HCN RS samples are presented in Fig. 3. Rice RS had the characteristics of A-type crystallinity with strong reflection peaks at about 15° and 23°, and an unresolved doublet at around 17° which was in conformity with the characteristics of normal cereal starches^55^. The XRD pattern of horsechestnut RS also showed the same characteristic peaks at 15° and a feeble peak at 23° which is in conformity with the results reported in literature^56^. The relative crystallinity of the rice RS varied from 16.75 (SK 406) to 17.72% (SK 46) however the lowest relative crystallinity was shown in horsechestnut (13.90%). The relative crystallinity of all rice and HCN RS were lower than the native rice and HCN^11,56^. This might be because of their higher amylose contents as amylose disrupts the crystalline packing of amylopectin^57^.

## Conclusion

In this study RS was extracted from the five Himalayan rice cultivars and HCN using porcine pancreatin enzyme following which it was subsequently characterized for its physicochemical and structural properties. *In vitro* digestibility test showed that RS content ranged significantly between the rice cultivars and HCN, ranging between 85.5 to 92.7% for rice and 99.5% for HCN. The RS extracted from the rice cultivars and HCN showed significant difference in the apparent amylose content (AAC), ranging between 31.83 to 40.68% for rice and 45.79% for HCN. Water absorption capacity (WAC), swelling and solubility index of RS ranged from 112–133.9%, 5.28–7.25 g/g and 0.033–0.044 g/g, respectively. The rice RS granules were polyhedral and irregular shaped with granular length in the range of 4.8–5.9 µm. The HCN RS granule morphology showed smooth surfaced round, elliptical, irregular and oval shapes with average granular length of 21 µm. Pasting properties also varied significantly between rice RS and HCN RS with the later showing lesser values. Thermal properties (T_o_, T_p_, T_c_) and ΔH gel also varied considerably between the rice RS and HCN RS, wherein the highest values for peak gelatinization temperature and gelatinization enthalpy were seen for CH-1039. X-ray diffraction of rice and HCN RS showed the A type of crystallinity pattern. Physicochemical and structural parameters of the rice and HCN RS successively revealed the potential of the extracted RS to be used for various functional purposes. HCN might serve as an efficient source for RS extraction as presently it goes waste. It was also observed from the above study that HCN has high amylose content and low swelling power than rice RS hence best suited to maintain the texture of foods.
